# Expansion of CD8^+^ T cell population in Lassa virus survivors with low T cell precursor frequency reveals durable immune response in most survivors

**DOI:** 10.1371/journal.pntd.0010882

**Published:** 2022-11-28

**Authors:** Stephanie M. LaVergne, Saori Sakabe, Mambu Momoh, Lansana Kanneh, Nell Bond, Robert F. Garry, Donald S. Grant, Juan Carlos de la Torre, Michael B. A. Oldstone, John S. Schieffelin, Brian M. Sullivan

**Affiliations:** 1 Viral-Immunobiology Laboratory, Department of Immunology and Microbiology, Scripps Research, San Diego, California, United States of America; 2 Division of Infectious Diseases, University of California, San Diego, California, United States of America; 3 Viral Hemorrhagic Fever Program, Kenema Government Hospital, Kenema, Sierra Leone; 4 Ministry of Health and Sanitation, Freetown, Sierra Leone; 5 Eastern Technical University of Sierra Leone, Kenema, Sierra Leone; 6 Department of Immunology and Microbiology, Tulane University School of Medicine, New Orleans, Louisiana, United States of America; 7 College of Medicine and Allied Health Sciences, University of Sierra Leone, Freetown, Sierra Leone; 8 Department of Pediatrics, Tulane University School of Medicine, New Orleans, Louisiana, United States of America; 9 La Jolla Institute for Immunology, San Diego, California, United States of America; NIAID Integrated Research Facility, UNITED STATES

## Abstract

**Introduction:**

Lassa virus is a priority pathogen for vaccine research and development, however the duration of cellular immunity and protection in Lassa fever (LF) survivors remains unclear.

**Methods:**

We investigated Lassa virus specific CD8^+^ T cell responses in 93 LF survivors. Peripheral blood mononuclear cells from these individuals were infected with recombinant vesicular stomatitis virus encoding Lassa virus antigens and virus specific T cell responses were measured after 18-hour incubation. Participants who had undetectable CD8^+^ T cell response underwent further analysis using a 10-day T cell proliferation assays to evaluate for low T cell precursor frequency.

**Results:**

Forty-five of the 93 LF survivors did not have a Lassa virus specific CD8^+^ T cell response. Of those with responses and a known date of onset of LF (N = 11), 9 had LF within the last ten years. Most participants without a measurable CD8^+^ T cell response were more than 10 years removed from a clinical history of LF (N = 14/16). Fourteen of 21 patients (67%) with undetectable CD8^+^ T cell response had a measurable Lassa virus specific CD8^+^ T cell response with the 10-day assay.

**Discussion:**

Despite reports of strong CD8^+^ T cell responses during acute Lassa virus infection, circulating Lassa virus-specific CD8^+^ T cells declined to undetectable levels in most Lassa fever survivors after ten years when evaluated with an 18-hour T cell stimulation. However, when Lassa virus-specific T cells were expanded prior to restimulation, a Lassa virus-specific CD8^+^ T cell response could be detected in many if the samples that were negative in the 18-hour stimulation assay, suggesting that prolonged cellular immunity does exist in Lassa fever survivors at low frequencies.

## Author summary

Lassa virus is estimated to infect several thousand people yearly in West Africa, resulting in many cases of Lassa fever disease associated with high morbidity and lethality. We found that durable cellular immunity exists in many Lassa fever survivors. Though larger studies are needed, these data could help shape vaccine research with a goal of lasting protection with immunization.

## Introduction

Lassa virus (LASV) is estimated to infect several thousand individuals across West Africa each year [1–4] resulting in many cases of Lassa fever (LF), a febrile disease associated with high morbidity and significant mortality [5,6]. Hospitalized cases of LF are a small percentage of total cases of LASV infection, but the lack of approved vaccines or interventions other than supportive therapy contribute to high fatality rates of around 20% in hospitalized patients [7–12]. The high case fatality rate in hospitalized patients and lack of prophylactic or preventative therapies have led to the categorization of LF by World Health Organization as a high priority disease for vaccine research and development [13].

Several studies have documented a seroprevalence of LASV within Western Africa that is much higher than the reported cases of LF [14,15], suggesting that a large number of LASV infections are asymptomatic, which may reflect that the immune response can control infection and prevent severe disease in most infected individuals. Evidence indicates that protective immune responses against lethal doses of LASV in non-human primates (NHPs) are likely primarily cell mediated [16,17], which has important implications for vaccine development. Thus, an early and robust CD4^+^ and CD8^+^ T cell responses in LASV infected NHPs is associated with survival, whereas in NHPs that succumb to LASV infection, T cell responses are delayed and weak [14,16–19].

Studies of LF survivors have documented robust T cell responses during and shortly following LASV infection [20–22]. However, the durability of LASV immunity in infected individuals remain poorly characterized. LASV-specific IgG antibodies have been identified in people up to 40 years after their initial exposure to LASV [23]. Robust CD8^+^ T cell responses were detected for up to nearly two months following acute LASV infection in a patient cared for in the United States [22], but there is very limited knowledge about the long term cellular immune response to LASV in humans. To fill this knowledge gap, we sought to evaluate the durability of LASV specific T cell responses in a cohort of LF survivors.

## Methods

### Ethics statement

This study was reviewed and approved by both Scripps Research and Tulane University Human Research Protection Program, and the Sierra Leone Ethics and Scientific Review Committee. Research was conducted according to the principles of the Declaration of Helsinki. All subjects provided written consent.

### Study site and participants

Kenema Government Hospital (KGH) in Kenema, Sierra Leone was the site of our study, and is the Ministry of Health and Sanitation, Government of Sierra Leone referral hospital for Kenema District. Participants were enrolled in a Lassa Survivor Cohort if they had previously been diagnosed with LF and hospitalized at KGH.

### Recombinant single cycle infectious vesicular stomatitis virus (rscVSV) preparation and T cell assays

We evaluated LASV-specific T cell responses by using recombinant single-cycle infectious vesicular stomatitis virus (rscVSVs) encoding either LASV nucleoprotein (NP), glycoprotein 1 (GP1), or glycoprotein 2 (GP2) constructed from the Josiah strain, as well as enhanced green fluorescent protein (eGFP) as a negative control. This method has been used by us and others to identify virus-specific CD4^+^ and CD8^+^ T cell responses to several pathogens [20,21,24–26]. Blood was collected at KGH, PBMCs were isolated, and stored at -80°C as previously described [24,25]. For T cell stimulation and quantification of LASV specific T cell responses, 18-hour and 10-day stimulation assays were completed. For the 18-hour assays, PBMC’s from LF survivors were infected with rscVSVs encoding full length LASV proteins NP, GP1, GP2, and sspGP2, at an MOI of 15. As a negative control, PBMCs were infected with rscVSV encoding green fluorescent protein (GFP), and unstimulated cells were also evaluated. Cells were incubated with anti-human CD3 (60 μg/mL) and CD28 (20 μg/mL) antibodies (BioXcel, New Haven CT) as a positive control. PBMCs were incubated at 37°C/5% CO_2,_ Brefeldin A (4 μg/mL) was added after 4 hours, then cells continued incubation overnight. Cells were subsequently stained with FITC-conjugated anti-human CD3 (BioLegend, San Diego, CA), PerCP-Cy5.5-conjugated anti-human CD4 (BioLegend, San Diego, CA), and PE-conjugated anti-human CD8 (BioLegend, San Diego, CA) antibodies at a dilution of 1:200 for 1 hour at 4°C. Cells were washed twice with FACS buffer (PBS containing 2% fetal bovine serum), then fixed with BD Cytofix buffer (BD Biosciences, Franklin Lakes, NJ) for 20 minutes at 4°C. After fixation, cells were washed twice with BD Perm/Wash buffer, then stained with PECy7- conjugated anti-human IFN-γ (BD Biosciences, Franklin Lakes, NJ) and APC-conjugated anti-human TNF-α (BD Biosciences, Franklin Lakes, NJ) antibodies diluted in BD Perm/Wash buffer for 1 hour, at 4°C. Cells were washed with BD Perm/Wash buffer twice, and suspended in FACS buffer in preparation for flow cytometry analysis. LSR II (Beckton Dickenson, Franklin Lakes, NJ) was used for flow cytometry data collection, and data was analyzed using FlowJo software (TreeStar, Woodburn, OR). The flow cytometry gating strategy included live lymphocytes, single cells, CD3^+^ cells, CD8^+^ or CD4^+^ cells, and proliferated cells in the analysis (S1 Fig). Cells were considered activated and with a positive response if the T cells were positive for both IFN-γ and TNF-α at 1.2log_10_ greater than the base population of T cells.

For the 10-day assay, PBMCs from LF survivors were incubated at 37°C with cell trace violet at a concentration of 1 uL per 4x10^6^ cells for 20 minutes, resuspended in RPMI and plated in a 96-well plate. Cells were then infected (MOI = 10) with rscVSVs encoding full length LASV proteins NP, GP1, GP2, and sspGP2, or GFP as a negative control. Positive controls were obtained as in the 18-hour assay, as described above. IL2 and IL15 (PeproTech Inc, East Windsor, NJ) were added to the PBMCs, each at a concentration of 0.02 ng/uL. PBMCs were incubated for 9 days at 37°C/5% CO_2_. On day 9 post infection, PBMCs were reinfected (MOI = 15) with rscVSVs, incubated for 4 hours at 37°C, and brefeldin A was added at a concentration of 4 μg /mL. PBMCs were incubated overnight at 37°C. Cells were then stained and prepared as in the 18-hour assay, as described above. In the tetramer assays, APC-conjugated tetramer was also used (HLA-A*02:01, NIH Tetramer Core Facility).

### Anti-LASV GP and anti-LASV NP antibody ELISAs

IgG seropositivity was assayed with pan-LASV NP and GP IgG ELISA plates (Zalgen Labs, Germantown, MD) which were prepared following the manufacturer’s instructions. Briefly, samples and provided negative controls were diluted 1:100 in sample diluent. For both NP and GP ELISAs, a 6-point reference curve using 3-fold serial dilutions was prepared using provided calibrators. All controls and samples were plated in duplicate and incubated at room temperature for 30 minutes. Following the 30-minute incubation, plates were washed using a 5-wash program (Biotek, 405Select) in provided ELISA wash buffer. Plates were incubated with IgG anti-human HRP-conjugated antibody solution for 30 minutes, followed by another washing step in ELISA wash buffer. The one-component substrate (TMB) was then added, and plates were incubated at room temperature for 10 minutes before the addition of the provided stop solution. Plates were read at 450 nm (Biotek, Synergy H1) and were considered positive if the resultant calculated concentration was above 1.65mg/mL (sensitivity: 92.6%, specificity: 95.2%) and 3.04mg/mL (sensitivity: 61.5%, specificity: 95.2%) for NP and GP IgG, respectively. Negative cutoffs were determined using an ROC analysis with a panel of sera from 122 known LF survivors and 130 individuals from a LASV non-endemic region in South America. LASV-IgG data was analyzed using 4-parameter logistic regression in Graphpad/PRISM version 9 (Carlsbad, CA). P values were calculated with one-way ANOVA, Tukey’s multiple comparisons test using Graph Pad Prism software.

### Plaque reduction neutralization tests

One hundred plaque forming units of low-passage LASV, Josiah strain, was incubated for 60 minutes with serial dilutions of patient sera to assess for neutralizing activity in Earle’s Minimal Essential Medium, 1% penicillin/streptomycin, and 1x glutamax with 10% guinea pig complement (Rockland, Limerick, PA). After incubation, sera-LASV mixture were plaque-assayed to measure residual infectivity. Vero 76 cells were inoculated with sera for 60 minutes, inoculum was then removed, and an agar medium was placed on the cells. The cells were then incubated for 6 days, then stained with neutral red for 24 hours. Plaques were measured and a 4-parameter logistic regression analysis was used to determine titers.

## Results

### LASV-specific CD8^+^ T cell responses using 18-hour assay

**Fig 1:**
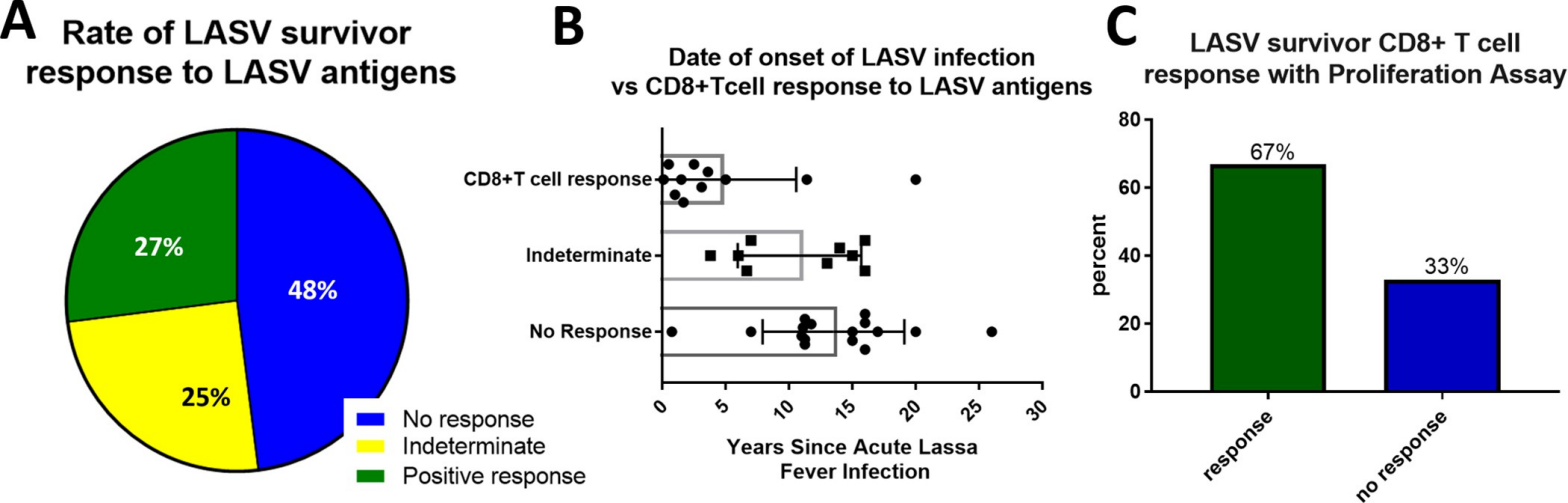
**A** Rate of CD8^+^ T cell response to LASV antigens. N = 93, including all LF survivors (with and without known date of infection); Responses were considered indeterminate if the LASV specific T cell response was present but background events in the negative controls and stimulated samples was high. **B** CD8^+^ T cell responses to LASV specific antigens in overnight assays in those with known date of onset of LASV infection (total N = 36); **C** Rate of response of LASV to proliferation assay, that were non-responders to standard overnight stimulation.

We have documented the identification of LASV-specific T cell epitopes by incubating PBMCs from LF survivors overnight with rscVSVs encoding LASV antigens [20,21]. We expanded this previous work to 93 LASV survivors and evaluated their CD8^+^ T cell responses to LASV antigens by stimulating PBMCs with rscVSVs encoding LASV NP and GPC sequences. Despite success using this method for evaluating Ebolavirus specific T cells in long-term survivors of ebolavirus disease (EVD) [24,25], only 27% of long-term LF survivors had a measurable CD8^+^ T cell response (Fig 1A) using this method. We considered LF survivors as having a positive T cell response if the percentage of T cells coexpressing IFN-γ and TNF-α was higher than both unstimulated and rscVSV-eGFP stimulated negative controls. Responses were considered indeterminate if the LASV specific T cell response was present but background events in the negative controls and stimulated samples was high.

We hypothesized that the inability to detect T cell responses in the majority of LF survivors was due to contraction of circulating LASV-specific T cells over time. However, unlike with EVD survivors, most LF survivors did not have well documented disease history due to poor record keeping during and just after the civil war in Sierra Leone. Consequently, the date of LASV infection onset was known for only 36 survivors, of which 31% had no CD8^+^ T cell response to overnight stimulation with LASV specific antigens (Fig 1B). Among LF survivors with positive T cell responses after overnight stimulation with rscVSVs, 81% had a date of LF onset within 10 years from the date of blood collection (N = 11). In contrast, onset of disease in survivors without a CD8^+^ T cell response was greater than 10 years in 87% of participants (N = 16) (p = 0.0008).

### LASV-specific CD8^+^ T cell responses using 10-day assay

Our data suggested that LASV-specific T cells may decrease over time or that our overnight stimulation assay was not sensitive enough to detect low precursor frequency LASV-specific T cells. We consequently altered our assay to increase sensitivity to detect lower frequency virus-specific T cells in LF survivors with no measurable T cell response. This was accomplished by an initial stimulation with our rscVSVs for 9 days in the presence of IL-2 and IL-15. We then performed an overnight restimulation with the homologous rscVSVs in the presence of brefeldin A and measured the expression of IFN-γ and TNF-α by flow cytometry.

The rscVSVs encoding LASV antigens used in this study have been described [20,21,24,27]. To avoid rescuing a replication competent VSV, we split the GP into GP1 (GPC: 1-279aa) and GP2 (GPC: 214–491) with an overlapping region to ensure that epitopes spanning this area would not be missed. Further, because the GP2 construct did not yield a stable protein, which might compromise detecting GP2-specific T cell responses, we rescued a rscVSV encoding a SSP-GP2 fusion protein (GPC: 1–59, 260–491) which was previously shown to be detected by Western blot [21]. In addition, we used a rscVSV encoding eGFP as a negative control. We did not include LASV Z and L proteins in our studies because compelling evidence indicates that most of the dominant T cell responses against mammarenaviruses recognize NP and GPC-specific epitopes [28–32].

Initially, 93 LF survivors were analyzed for LASV specific CD8^+^ T cell responses with an 18-hour stimulation assay, and 45 did not respond [20,21]. Participant characteristics for all 93 survivors is shown in Table 1.

**Table 1:** Participant characteristics.

Participant characteristics	All participants N = 93	With CD8^+^ T cell response to 18h assay N = 25	Without CD8^+^ T cell response to 18h assay N = 45	With Indeterminate CD8^+^ T cell response to 18h assay N = 23
**Age in years, mean (Range)**	32.3 (7–70)	24.7 (7–43)	33.3 (8–70)	38.2 (23–60)
**Female Sex, no (%)**	59 (63%)	17 (68%)	25 (55%)	17 (74%)
Years from LASV infection to data collection, mean (Range)* **†**	9.5 (1–26)	4.5 (1–20)	12.9 (1–26)	9.5 (4–14)

*Date of LASV infection was known for 36 of 93 participants

**†** those without CD8^+^ T cell responses to the 18-h assay had more remote LASV infections than those with CD8^+^T cell responses (p = 0.001)

We had enough sample to complete T-cell proliferation assays in 21 of these 45 non-responders. Twenty-one survivors who had no CD8^+^ T cell response to overnight stimulation with LASV specific antigens were subsequently stimulated using this 10-day proliferation and restimulation assay. Of the 21 participants, 43% were female (Table 2). Participants’ age range was 18–55 years, with similar mean ages in those with and without T cell responses following expansion and stimulation. Individuals without a T cell response had an older onset of LF disease than those with T cell responses (Table 2).

**Table 2:** Participant characteristics.

Participant characteristics	All participants N = 21	With CD8^+^ T cell response to 10-day assay N = 14	Without CD8^+^ T cell response to 10-day assay N = 7	P value
**Age in years, mean (Range)**	33.2 (18–55)	33 (18–55)	33.8 (25–45)	0.89
**Female Sex, no (%)**	9 (43%)	6 (43%)	3 (43%)	1
**Years from LASV infection to data collection, mean (Range)**	14 (4–28)	12.4 (4–20)	22.5 (17–28)	0.29

**Fig 2:**
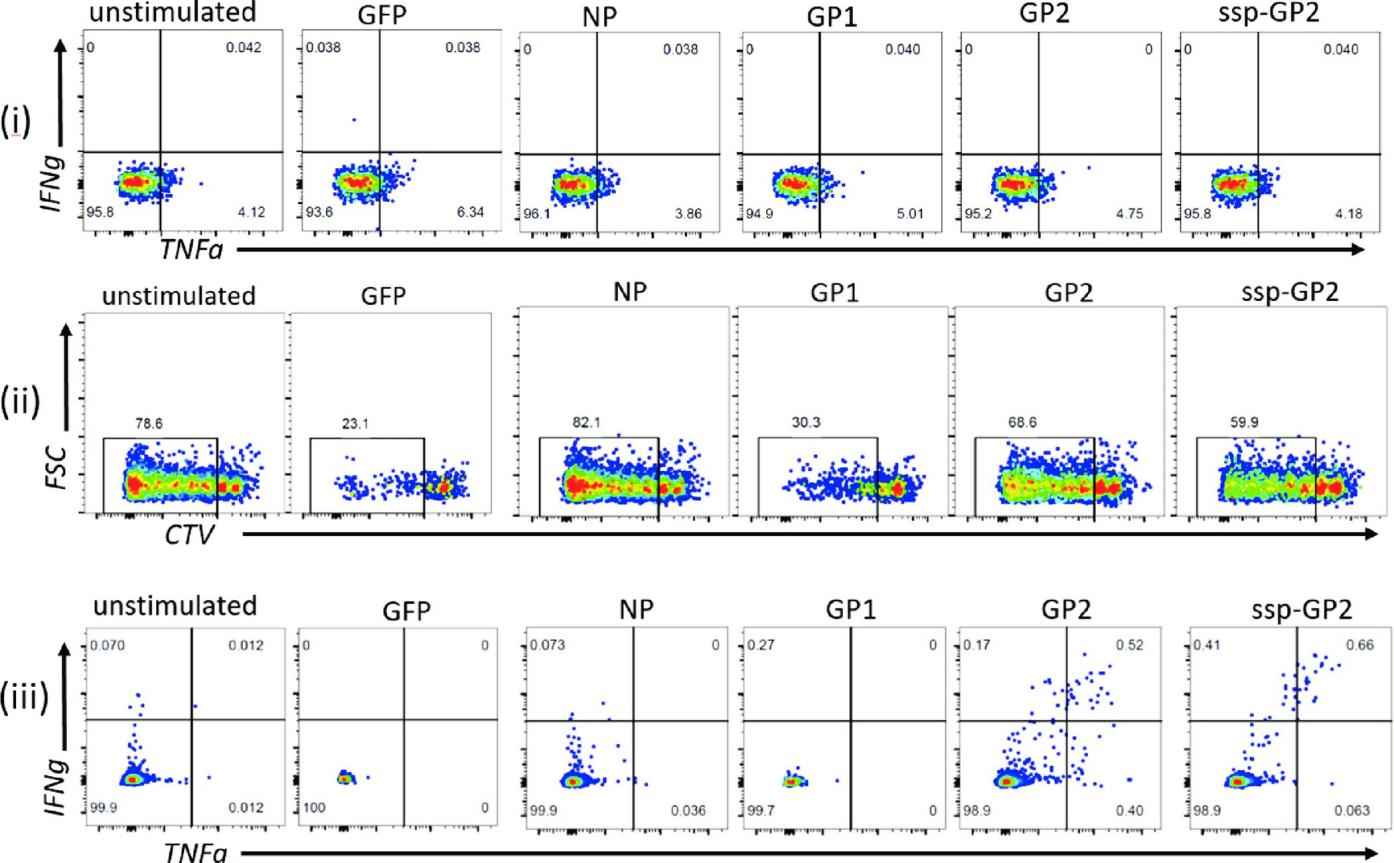
(i) A LF survivor with a negative LASV-specific T cell response to overnight stimulation with different antigens, as denoted above each plot. The same survivor’s proliferated CD8^+^ T cells (ii) have a positive LASV-specific response (iii) to a prolonged stimulation 10-day proliferation assay.

**Fig 3:**
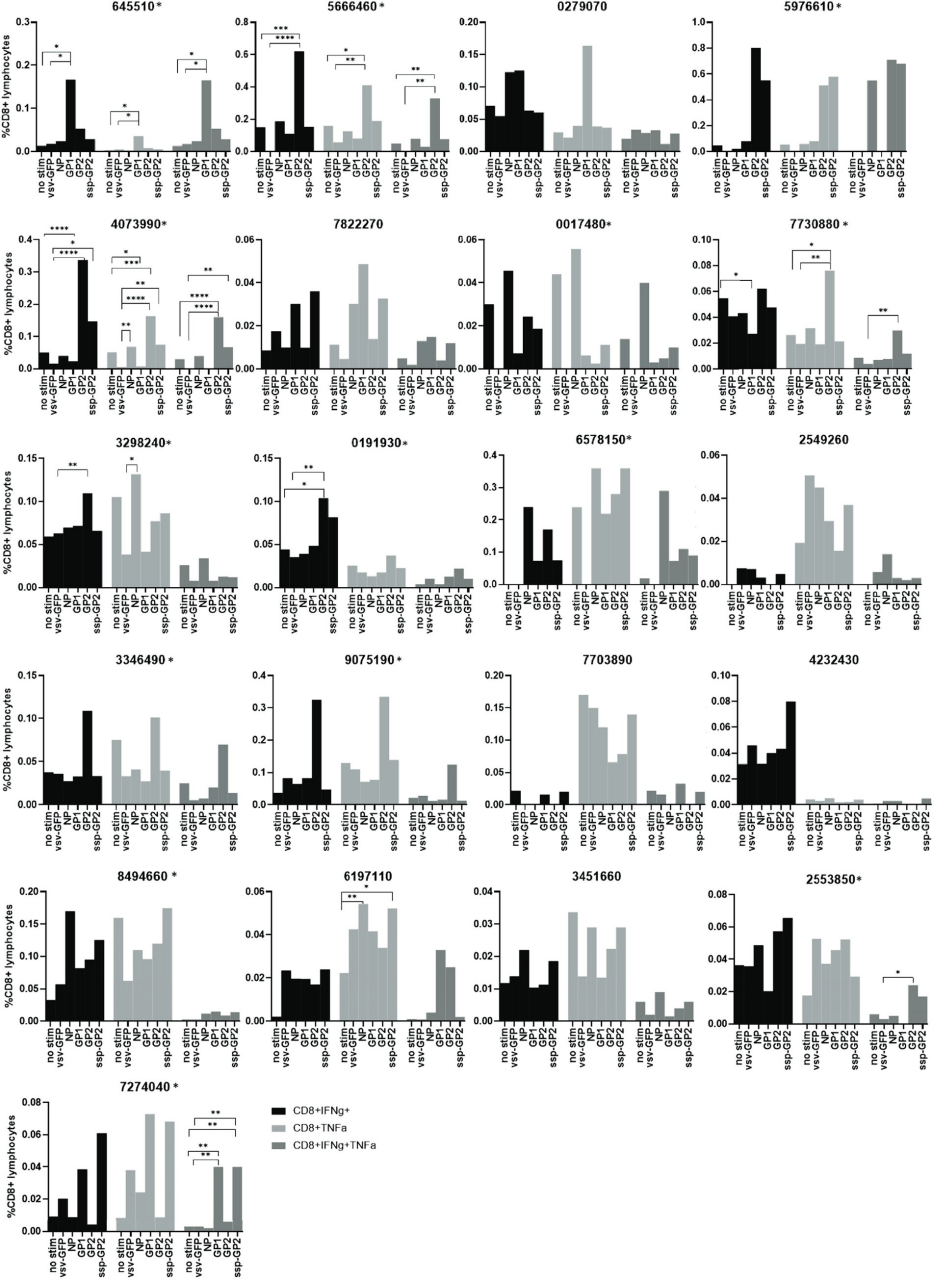
Individual LF survivors’ CD8^+^ T cell response, measured by percentage of T cells expressing IFN-γ alone, TNF-α alone, or both IFN-γ and TNF-α, when stimulated by LASV antigens and negative controls (negative controls include no stimulation and GFP). Asterisk on individual plots indicates the 14 LASV survivors with positive IFN-γ+ TNF-α+ CD8 T cell response. 3 replicates were performed for 645510, 5666460, 4073990, 7822270, 0017480, 7730880, 3298240, 0191930, 2549260, 3346490, 4232430, 6197110, 3451660, 2553850, 7274040. Due to limited sample, 2 replicates were performed for 0279070, 9075190, 6578150 and 8494660 and 1 replicate was performed for 5976610 and 7703890.

CD8^+^ T cell responses were measured by co-expression of IFN-γ and TNF-α. Fourteen of the 21 survivors (67%) who had no CD8^+^ T cell response to the overnight stimulation assay had a IFN-γ +TNF-α+ CD8+ T cell response to the 10-day expansion and stimulation assay (645510, 5666460, 5976610, 4073990, 0017480, 7730880, 3298240, 0191930, 6578150, 3346490, 9075190, 8494660, 2553850, 7274040) (Fig 1C). An example of a LF survivor (5976610) without a response to the overnight assay and with a response to the 10-day proliferation assay is shown in Fig 2. Six of the 21 LF survivors examined had significantly higher T cell responses to LASV antigens when compared to the negative unstimulated or GFP controls (Fig 3). When compared to negative unstimulated and GFP controls, four survivors (2553850, 5666460, 4073990, 7730880) had significantly higher CD8^+^T cell response to LASV GP2 antigen, two survivors had significantly higher response to sspGP2 (4073990, 7274040), and two survivors had significantly higher GP1 response (645510, 7274040) (Fig 3). Four survivors had a good T cell response, but there were too few replicates to calculate statistical significance (5976610, 6578150, 9075190, 8494660). Four survivors had a trend towards increased CD8^+^ T cell responses to expansion and stimulation, but differences did not reach statistical significance (0017480, 0191930, 3346490, 2553850).

Single cytokine analysis was also done evaluating CD8^+^IFN-γ^+^ and CD8^+^TNF-α^+^ cells (Fig 3), following expansion and stimulation with LASV antigens. IFN-γ levels were statistically higher than negative controls in 5666460, 4073990, 3298240, and 0191930 when stimulated with GP2; 4073990 when stimulated with sspGP2; and 645510, 4073990, 7730880, and 5666460 when stimulated with GP1 (Fig 3). TNF-α levels were statistically higher than unstimulated controls in 5666460, 4073990, and 7730880 when stimulated with GP2; 4073990 and 6197110 when stimulated with sspGP2; 645510, 4073990 and 6197110 when stimulated with GP1; 3298240, 4073990, 3298240, and 6197110 when stimulated with NP (Fig 3).

**Fig 4:**
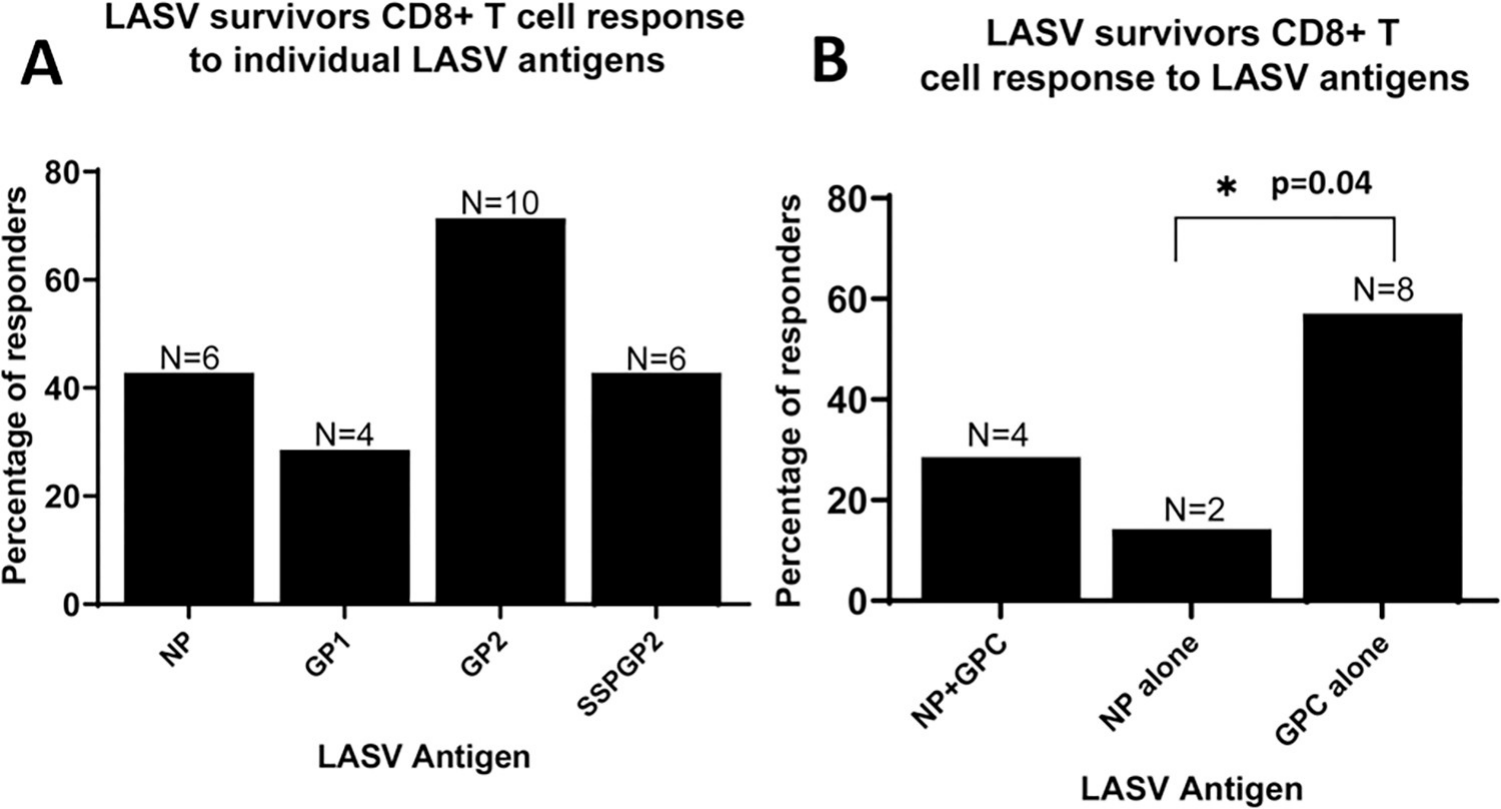
**A** Frequency of CD8^+^ T cell response to individual LASV antigens in LF survivors, excluding those that did not respond to the proliferation assay. **B** Frequency of CD8^+^ T cell response to either NP + GP, NP alone or GP alone, excluding non-responders.

Most survivors showed a response to GP2 (10 of the 14 responders), next to NP or sspGP2 (6 of the 14 responders each), then GP1 (4 of the 14 responders) (Fig 4A). Four of the survivors with CD8 T cell response had a response to NP and GPC antigens, two had a response to NP alone, and eight had a response to GPC alone (Fig 4B).

We also evaluated CD4^+^ T cell responses among the 21 LF survivors who had no response to the overnight T cell assays. Of these, four (3298240, 191930, 4232430, and 3451660) had a significantly higher IFNg^+^TNFa^+^CD4^+^ T cell response than negative unstimulated or GFP stimulated controls (S2 Fig). Participant 3298240 had CD4^+^ T cell responses to the GP2 antigen that were significantly higher than the control (p = 0.03). Participant 0191930 showed significantly higher responses to NP and GP2 antigens than the controls (p = 0.004 and 0.003, respectively). 4232430 and 3451660 both had significantly higher responses to NP and sspGP2 than controls (S2 Fig).

### GPC tetramer stain and 10-day proliferation assay

**Fig 5:**
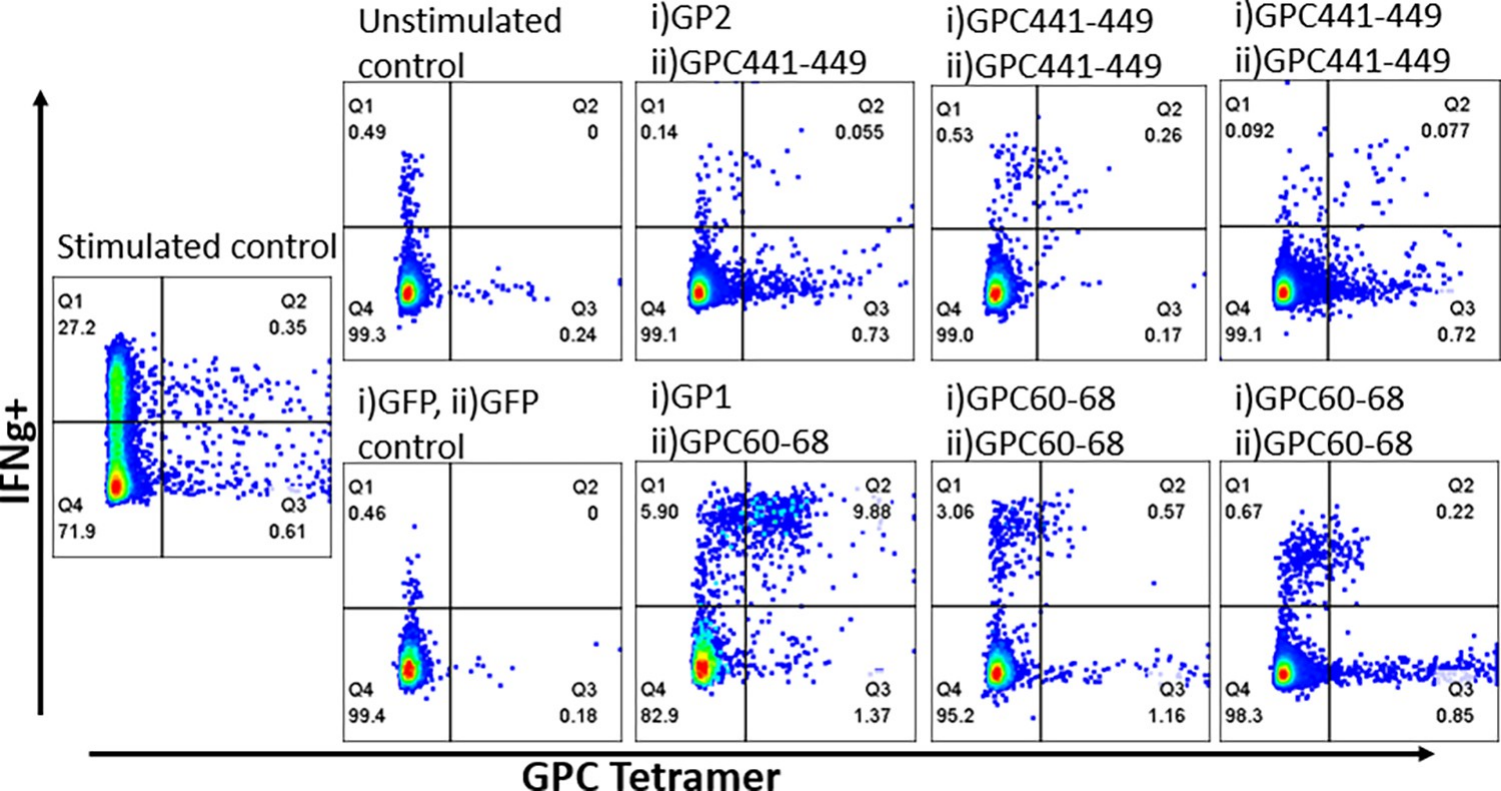
LF survivor’s CD8^+^ T cell responses to i) expansion and ii) stimulation, as denoted at the top of individual plots. On day 0, cells were expanded with either vsvGP2, vsvGP1, GPC441-449, or GPC60-68 peptides. Cells were then stimulated on day 9 with either GPC441-449 or GPC60-68 peptides. As a negative control, rscVSV-GFP was also used to expand and stimulate PBMCs. Staining included the GPC tetramer specific for the homologous peptide stimulation.

Because we had determined the HLA haplotypes of all our participants, we were able to selectively interrogate LASV peptide-specific CD8+ T cell responses in an individual harboring HLA A*02:01 allele [20,33]. As this survivor had a positive LASV-specific CD8^+^ response to the 18-hour T cell assay, we were able to use LASV GPC_60-68_ and GPC_441-449_ A*02:01 tetramers to evaluate the specificity of our results. We observed high levels of CD8^+^/Tetramer^+^/ IFN-γ^+^ cells, when compared to unstimulated and GFP stimulated controls (Fig 5). Similar findings were observed in GP1 stimulated or peptide stimulated cells.

### LASV anti-GP and anti-NP antibody levels

**Fig 6:**
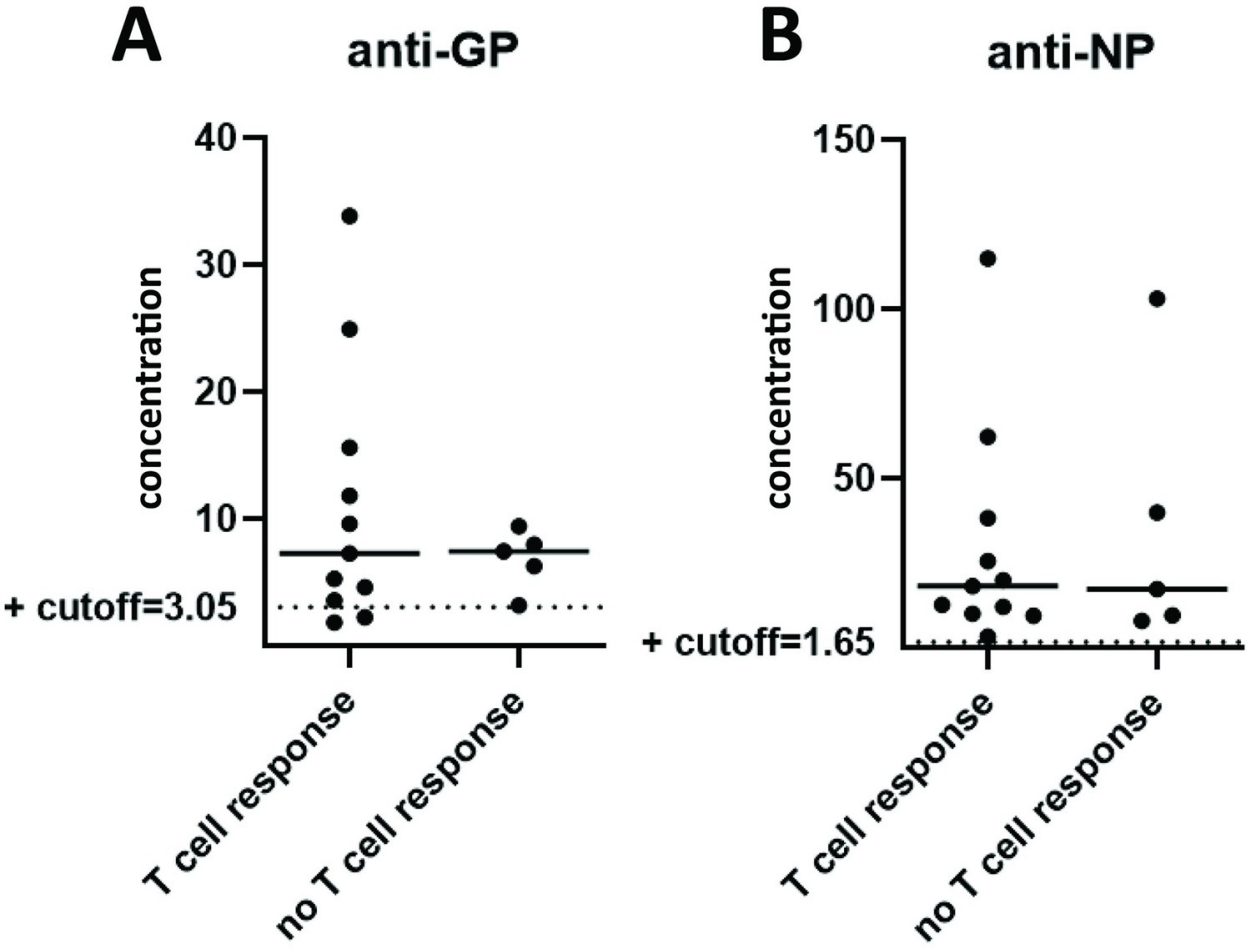
**A** Anti-GP and **B** anti-NP antibody levels in LF survivors with and without LASV-specific CD8^+^T cell responses on 10-day proliferation assay.

We sought to correlate T cell responses with LASV glycoprotein (GP) and nucleoprotein (NP) antibody levels. Of the 21 LF survivors with 10-day T cell assays completed, 16 had sera available for ELISA antibody testing. Fourteen samples had anti-GP antibody titers above the cutoff value (3.05). The mean anti-GP antibody value was 11.0 in LF survivors who had LASV specific CD8^+^ T cell responses, compared to 6.9 in those without a response (Fig 6A). Conversely, those with a LASV-specific CD8^+^ T cell response had a higher anti-NP response (mean = 35.6) vs those who had no T cell response (mean anti-NP = 29.7) (Fig 6B).

**Fig 7:**
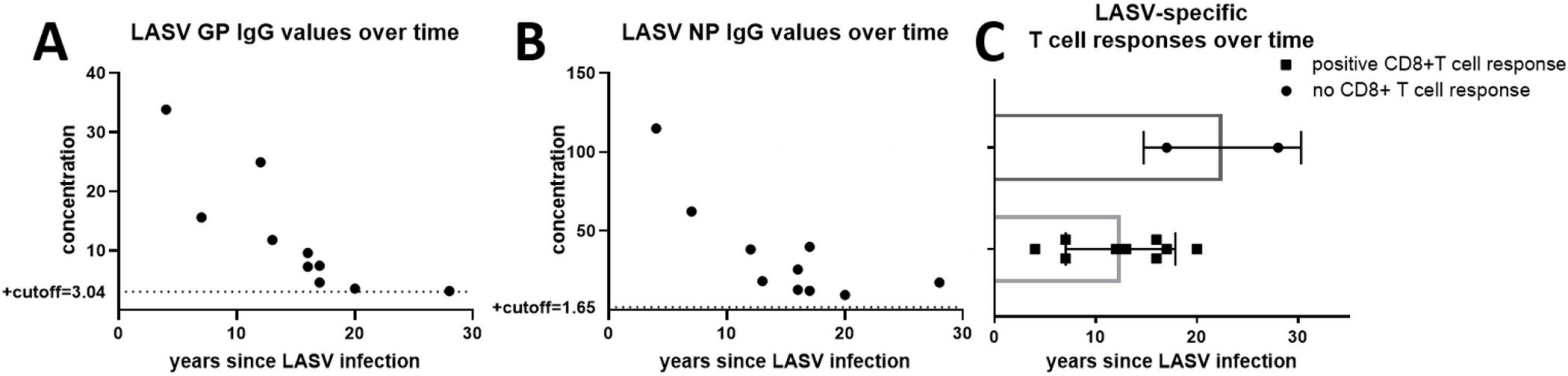
**A** and **B**) LASV GP and NP IgG values decrease over time from infection (N = 10) **C**) LASV specific T cell responses to 10-day proliferation assay decrease over time from infection (N = 11).

We also found that LASV GP and NP antibodies (Fig 7A and 7B) and T cell responses to the proliferation assay (Fig 7C) decreased over time in LF survivors with known date of LASV disease onset.

### LASV neutralizing antibody titers

**Fig 8:**
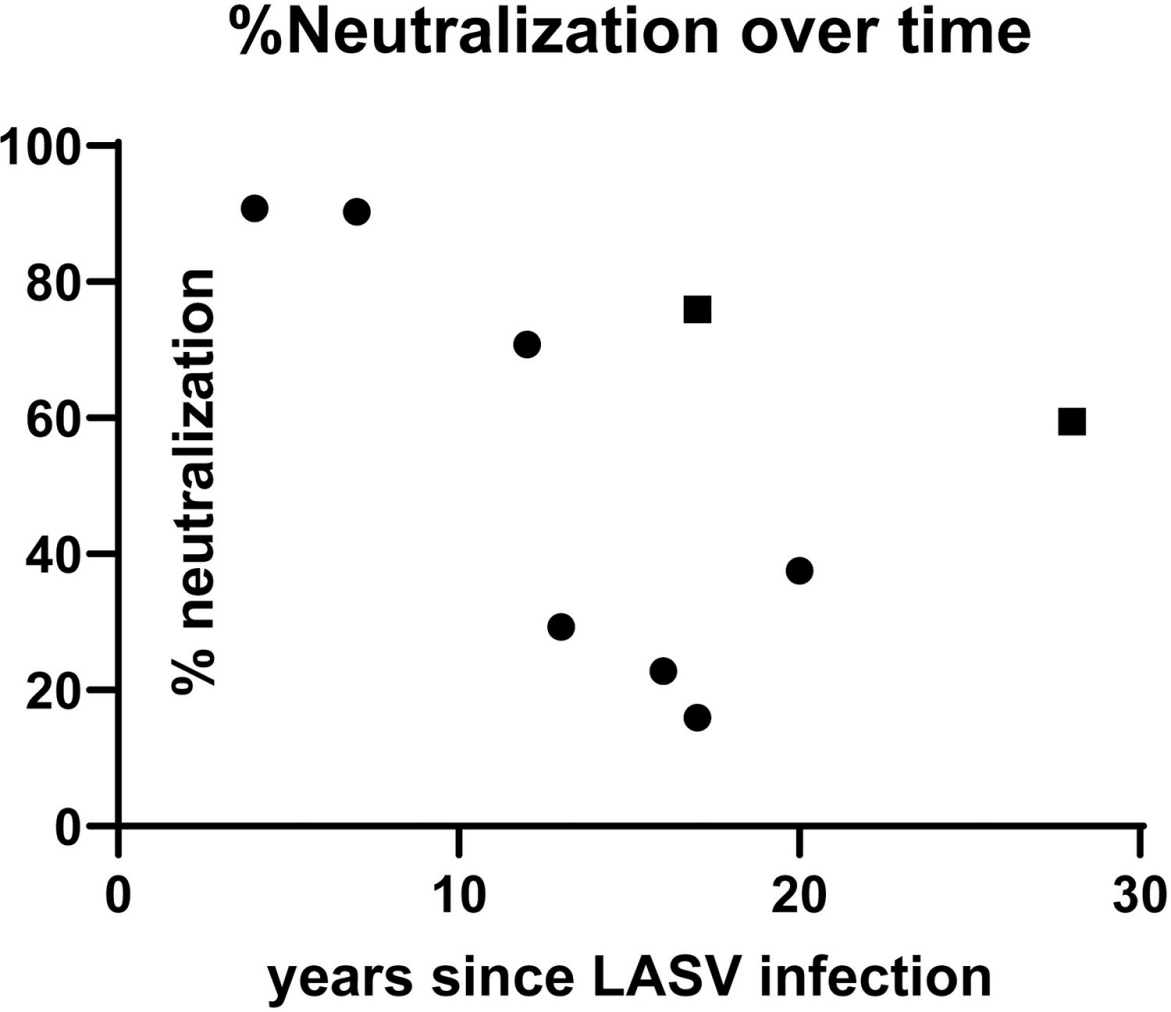
Percent LASV neutralization in LF survivors by years since LF infection. N = 9 Data points depicted as square symbols represent LASV survivors without T cell responses in 10 day expansion assays (N = 2).

Next, we evaluated neutralizing antibody titers in the 21 LF survivors with 10-day T cell assays completed. We had enough sample to perform neutralization assays for 9 survivors. Those who had positive CD8 T cell responses had an average percent neutralization of 51% (N = 7, ranging 15%-90%), and those without CD8 T cell responses had an average percent neutralization of 68% (N = 2, 59–75%) (Fig 8).

## Discussion

Sierra Leonean LF survivors have low rates of CD8^+^ T cell response to LASV antigens when PBMCs were stimulated in an 18-hour assay. Higher LASV-specific CD8^+^ T cell response rates were seen when PBMCs were initially stimulated with LASV rscVSV and allowed to expand in 10-day proliferation assays, even in LF survivors who were infected with LASV over 20 years prior to the assay. These findings show that durable cellular immunity exists in many LF survivors, though with low antigen-specific precursor T cell frequencies. CD4^+^ T cell responses to LASV specific antigens were also higher than in unstimulated or GFP stimulated cell populations, however the response was not as robust as seen with CD8^+^ T cells. Some survivors had higher IFN-γ and TNF-α levels measured in both CD8^+^ and CD4^+^ cell populations when compared to unstimulated or GFP stimulated cell populations, however higher background in single cytokine positive cells make it more difficult to accurately assess specificity of T cell activation compared to TNF-α^+^IFN-γ ^+^ T cells. One weakness of this study was that viability was not used, however we hoped that the use of GFP and unstimulated controls, as well as narrow gating strategies obviated the need for viability dye.

We found no correlation between anti-GP or anti-NP antibodies and LASV-specific CD8^+^ T cell responses. An outlier in the LF survivor group without CD8^+^ responses had a very high anti-NP response, which skewed the data in this small group and contributed to the absence of statistically significant differences, which reflected a major limitation in this study, namely the small size of PBMC samples available to us at the time of this study.

Though robust T cell responses have been observed during, and shortly after, LF, there is very limited information about the durability of LASV-specific T cell responses. Here we have shown LASV-specific T cell responses in LF survivors whose documented infections occurred twenty years prior. One limitation of our study is the inability to assess how often LF survivors are exposed to LASV. While repeated exposures could strengthen T cell responses over time, we observed a decrease in precursor frequency of T cell responses after approximately ten years. These data suggest that re-exposure is probably an uncommon occurrence in LF survivors.

Neutralizing antibody levels are not seen until several months after infection [1,34,35]. Little is known about the human T cell responses save a single case of non-fatal severe Lassa fever where robust T cell responses were observed early during symptomatic infection and remained robust for several weeks [22]. This observation is consistent with non-human primate studies of Lassa fever demonstrating that fatal Lassa fever was associated with poor or delayed T cell responses. Taken together, the absence of neutralizing antibodies at the time of viral clearance suggests that CTLs may play a stronger role during acute infection while neutralizing antibodies may contribute to long term immunity.

Survivors are thought to be protected against disease but not against reinfection [36]. Observations of anti-LASV antibodies over several decades and the long-term presence of LASV-specific T cells observed here suggest both may play a role in protection upon reinfection [23]. Even low precursor frequency of LASV-specific T cell responses may be amplified to respond and provide protection against recurrence of LF on re-exposure to the virus. Thus, engineering vaccine responses to induce similar durable protection may be an effective strategy for prevention of LF. A similar decline in cytotoxic T lymphocyte (CTL) responses that are robust with expansion have been observed with other viruses, including the measles virus [37–39]. The durability of T cell responses, especially to GP2, provide evidence that inclusion of GPC in vaccines designed to protect against LF may provide significant T cell immunity in addition to eliciting neutralizing antibody responses.

## Supporting information

S1 Fig Flowjo gating scheme. (TIF)

S2 Fig Individual LF survivors’ CD4^+^ T cell response, measured by percentage of T cells expressing IFN-γ alone, TNF-α alone, or both IFN-γ and TNF-α, when stimulated by LASV antigens and negative controls (negative controls include no stimulation and GFP). 3 replicates were performed for each sample.
(TIF)
