# Supplementary figures and images for: Expansion of CD8+ T cell population in Lassa virus survivors with low T cell precursor frequency reveals durable immune response in most survivors
Source: PLoS Negl Trop Dis. 2022 Nov 28;16(11):e0010882. doi: 10.1371/journal.pntd.0010882 (PMC9731491; doi:10.1371/journal.pntd.0010882)

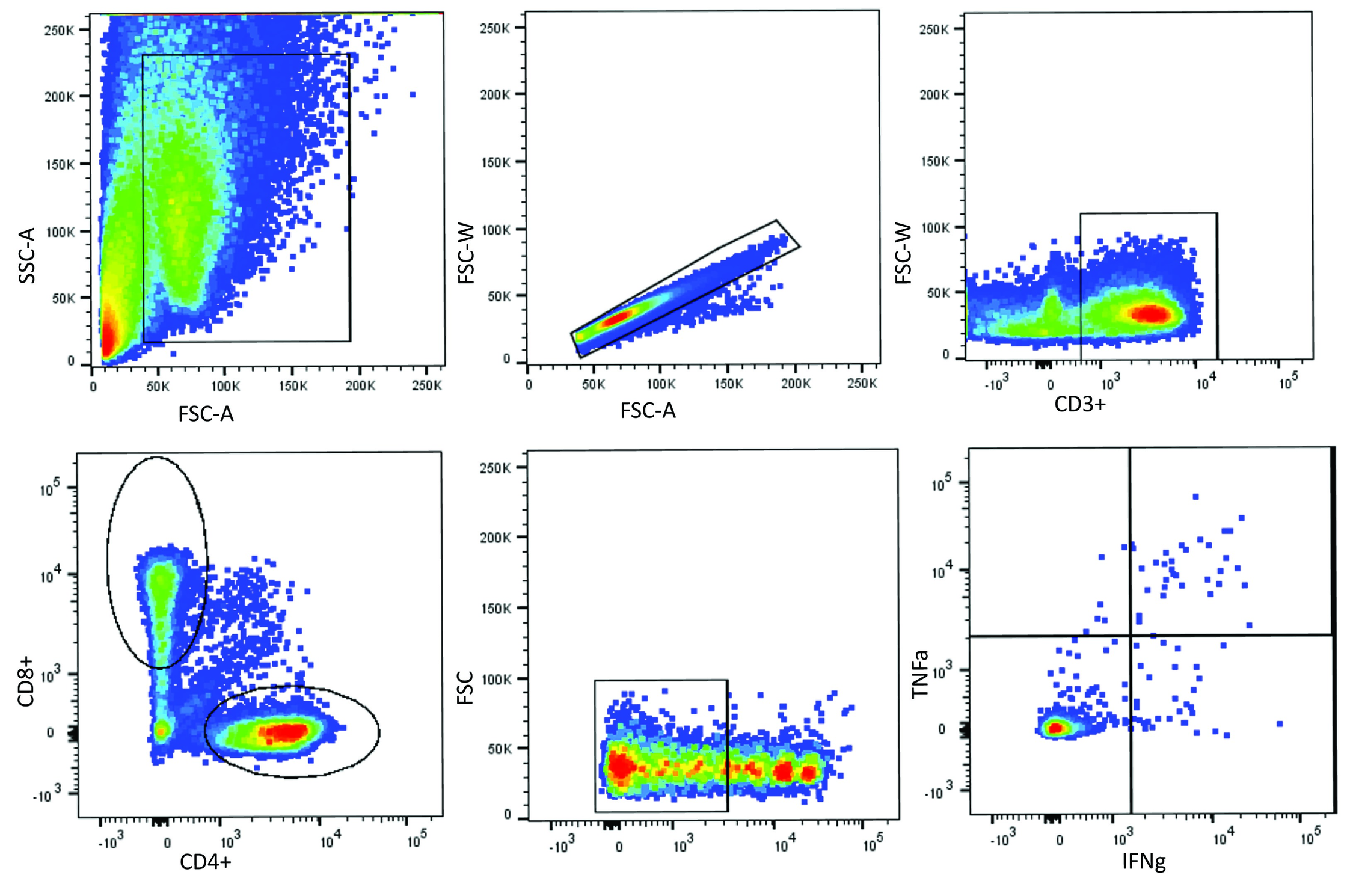

Supplement: S1 Fig — (TIF) [file pntd.0010882.s001.tif]

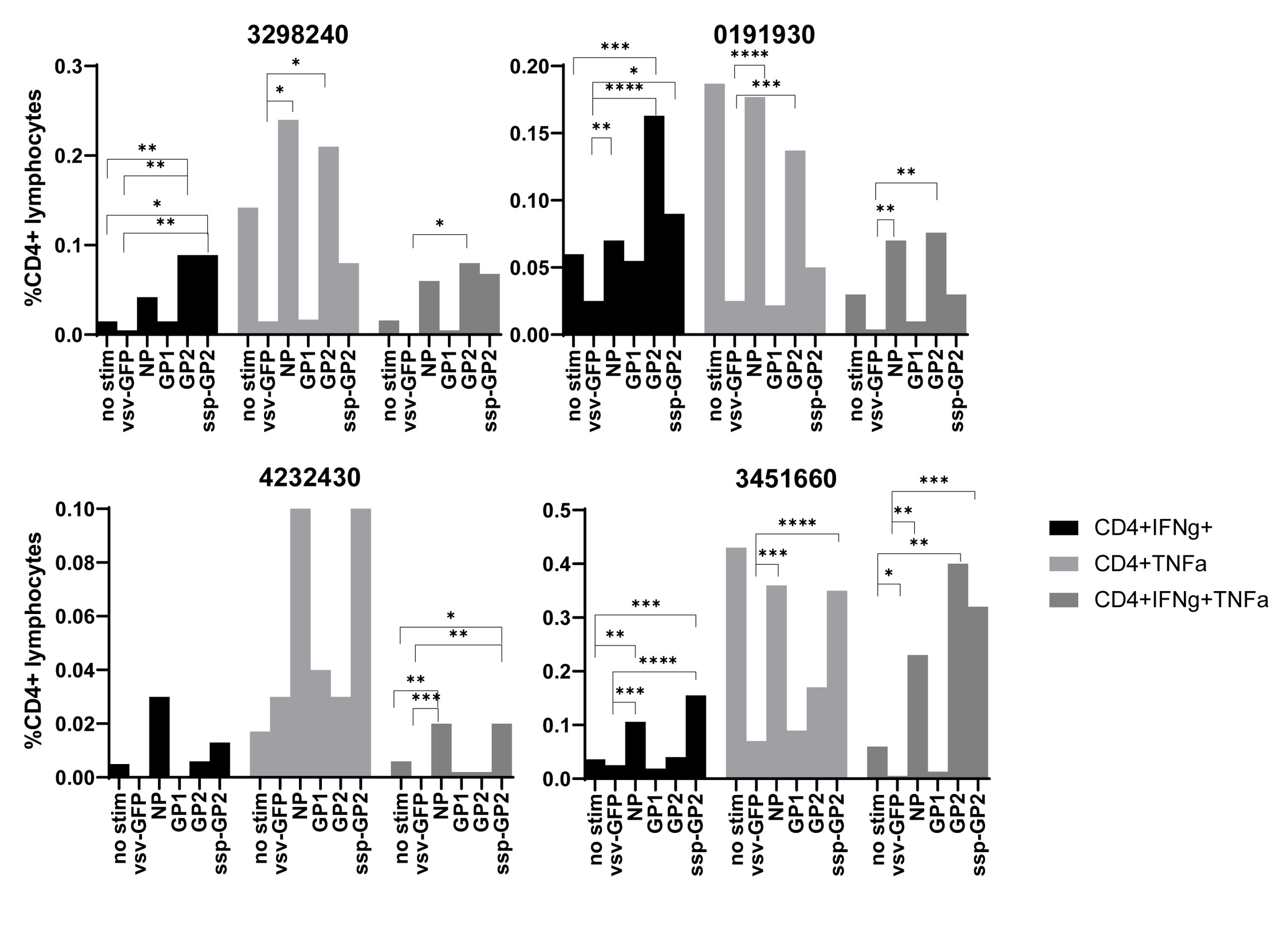

Supplement: S2 Fig — 3 replicates were performed for each sample. (TIF) [file pntd.0010882.s002.tif]
